# Maize *brachytic2* (*br2*) suppresses the elongation of lower internodes for excessive auxin accumulation in the intercalary meristem region

**DOI:** 10.1186/s12870-019-2200-5

**Published:** 2019-12-27

**Authors:** Xiangge Zhang, Xianbin Hou, Yinghong Liu, Lanjie Zheng, Qiang Yi, Haojun Zhang, Xinrong Huang, Junjie Zhang, Yufeng Hu, Guowu Yu, Hanmei Liu, Yangping Li, Huanhuan Huang, Feilong Zhan, Lin Chen, Jihua Tang, Yubi Huang

**Affiliations:** 1 State Key Laboratory of Crop Genetics of Disease Resistance and Disease Control, Sichuan Agricultural University, Chengdu, 611130 Sichuan China; 2 College of Agronomy, Sichuan Agricultural University, Chengdu, 611130 Sichuan China; 3 College of Agriculture and Food Engineering, Baise University, Baise, 533000 Guangxi China; 4 Maize Research Institute, Sichuan Agricultural University, Chengdu, 611130 Sichuan China; 5 College of Life Science, Sichuan Agricultural University, Ya’an, 625014 Sichuan China; 6 National Key Laboratory of Wheat and Maize Crop Science, Henan Agricultural University, Zhengzhou, 450000 Henan China

**Keywords:** Auxin efflux, *br2*, Dwarfism, Internode elongation, Intercalary meristem, Maize

## Abstract

**Background:**

Short internodes contribute to plant dwarfism, which is exceedingly beneficial for crop production. However, the underlying mechanisms of internode elongation are complicated and have been not fully understood.

**Results:**

Here, we report a maize dwarf mutant, *dwarf2014* (*d2014*), which displays shortened lower internodes. Map-based cloning revealed that the *d2014* gene is a novel *br2* allele with a splicing variation, resulting in a higher expression of *BR2-T02* instead of normal *BR2-T01*. Then, we found that the internode elongation in *d2014/br2* exhibited a pattern of inhibition-normality-inhibition (transient for the ear-internode), correspondingly, at the 6-leaf, 12-leaf and 14-leaf stages. Indeed, *BR2* encodes a P-glycoprotein1 (PGP1) protein that functions in auxin efflux, and our in situ hybridization assay showed that *BR2* was mainly expressed in vascular bundles of the node and internode. Furthermore, significantly higher auxin concentration was detected in the stem apex of *d2014* at the 6-leaf stage and strictly in the node region for the ear-internode at the 14-leaf stage. In such context, we propose that BR2/PGP1 transports auxin from node to internode through the vascular bundles, and excessive auxin accumulation in the node (immediately next to the intercalary meristem) region suppresses internode elongation of *d2014*.

**Conclusions:**

These findings suggest that low auxin levels mediated by BR2/PGP1 in the intercalary meristem region are crucial for internode elongation.

## Background

Dwarfism or semi-dwarfism confers a number of advantages to crop varieties, such as increased lodging resistance, denser growth, and higher harvest index, which are extraordinarily beneficial for crop production [1–3]. For crops, height is usually determined by the internodes’ number and length [4]. Numerous dwarf mutants have been characterized by short internodes, which have been attributed to impaired internode elongation. Therefore, increasing our knowledge of internode elongation will aid the improvement of crop yield.

Stem/internode elongation is mainly controlled by several plant hormones, including gibberellins (GAs), brassinosteroids (BRs), strigolactones (SLs), and auxins [5]. Defects in the biosynthesis or the signaling of these hormones can cause dwarf phenotypes [6–10], although each hormone might play a different role in stem/internode elongation, irregularities of which will lead to various mutant phenotypes. In maize, there are several GA-related mutants that display extremely reduced height with uniformed short internodes, such as *dwarf1*(*d1*), *d3*, *d5* and *anther ear1* (*an1*), as well as dominant mutants *D8* and *D9* [11–13], all of which influence internode elongation throughout the growth period, accompanied by a certain degree of yield loss. Most BR-associated mutants exhibit multiple defective phenotypes in addition to dwarfism [14–17]. For example, the maize *nana plant2* (*na2*) mutant displays suppression of tillers, altered leaf morphology, and andromonoecy [18]. Unlike GAs and BRs, the reduction in plant height of SL mutants may be an indirect effect of increased tillers because the deficiency in SLs enhances cell division in axillary meristems (AMs) [19, 20], which ultimately leads to a redirection of the nutrition toward tiller growth instead of internode elongation.

Auxin biosynthesis is performed through multiple Trp-dependent or Trp-independent pathways, mainly involving *YUCCA* (*YUC*) and *TAA1*/*TAR1*/*TAR2* genes [21]. Quadruple *yuc1 yuc4 yuc10 yuc11* mutants do not develop a hypocotyl and root meristem [22]. Similarly, *taa1* mutants in *Arabidopsis* displaye a defective hypocotyl and root [23] and *taa1 tar1 tar2* triple mutants lacked roots and were seedling lethal [24]. In maize, the *vanishing tassel2* (*vt2*) gene is an ortholog of *Arabidopsis TAA1*, and *vt2* mutants exhibit no tassel branches or spikelets, as well as a semi-dwarf phenotype with fewer leaves [25]. Many organs, including the stem/internode, are damaged in auxin biosynthesis mutants. The SCF^TIR1/AFB^-mediated proteolysis of Aux/IAA proteins is the major auxin signaling pathway, which is clearly responsible for many auxin actions [26, 27]. Most mutants in these components have a similar seedling lethal phenotype [28–30]. In addition, synthesized auxin is often directionally transported by auxin transporters to specific tissues, where it acts a potent signal that triggers a plethora of developmental responses [31]. The maize *br2* and sorghum orthologue *dwarf3* (*dw3*) exhibit reduced auxin transport and shortened statures [32], and auxin transport is crucial for regulating internode elongation.

Actually, several dwarf mutants described above exhibited various abnormal patterns of internode elongation and other morphological characteristics, which suggests that their underlying mechanisms should be discriminating and be worth pursuing further. In this study, we identified a novel *br2* allelic mutant, *d2014*, that exhibited shortened lower internodes but nearly normal upper parts, indicating that *br2* has a unique regulation on plant height development. The *br2* is a very famous dwarf gene (first cloned in 2003), which was considered ideal for shortening maize’s height due to its unique phenotype (mild dwarf, shorter lower internodes yet nearly normal upper internodes) [32, 33]. It was well-suited to dissect the mechanism of plant height development for maize improvement. Here, in order to reveal the effects of the *d2014*/*br2* mutation on internode elongation, we performed a dynamic comparison of internode elongation at several stages between *d2014* and wild type (WT) plant. Furthermore, we explored the specific location of *BR2* expression in the stem and detected the dynamic variation of auxin concentration so as to reveal the mechanism of internode elongation by BR2/PGP1-mediated auxin transport.

## Results

### Characterization of the maize *d2014* dwarf mutant

**Fig. 1:**
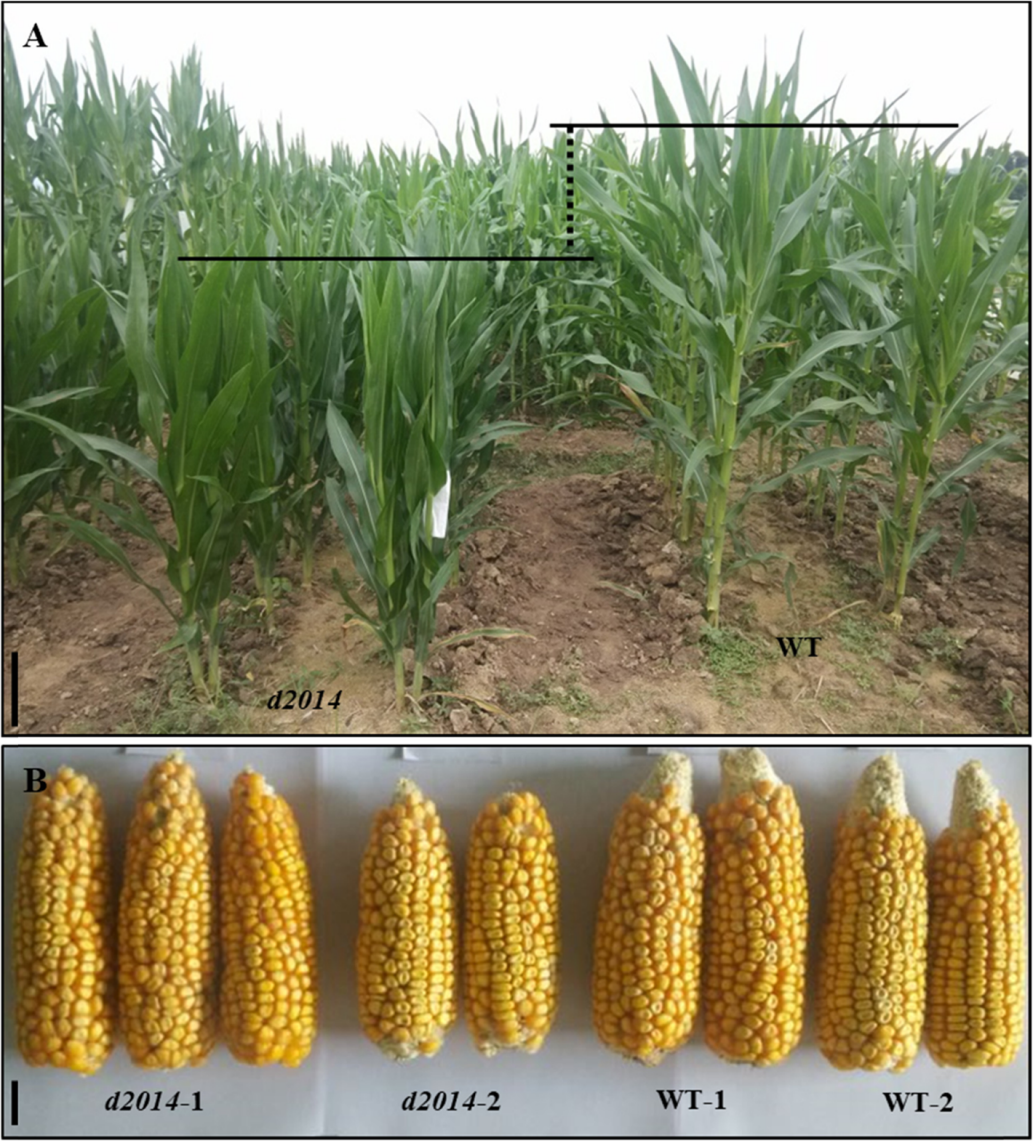
Morphological comparison between *d2014* and WT. **a** Plant morphology of *d2014* and WT in the field at the heading stage. The vertical dotted line indicates the difference of plant height between *d2014* and WT. *Bar* = 40 cm. **b** Ear morphology of *d2014* and WT at the mature stage. *Bar* = 2 cm

A maize dwarf mutant, *d2014*, arose spontaneously from the HL9047 (WT) inbred line in 2014, and its self-crossed progenies steadily presented with uniform short stature with erect leaves (Fig. 1a). The plant height of *d2014* was reduced by 58.61 cm, whereas its ear height was 44.59 cm less than that of WT (Table 1). This showed that the reduced height of lower internodes mainly contributes to the *d2014* dwarf phenotype. Additionally, other traits of *d2014*, such as ear length, ear width, ear weight, hundred-grain weight, total tassel length, tassel branch number, tasseling stage, and total leaf number were altered slightly in comparison with WT (Fig. 1b; Table 1). Especially for the ear traits, small variations were not enough to lead to yield loss. Therefore, the *d2014* mutant might be useful in maize breeding programs.

**Table 1:** Phenotypic comparison between *d2014* and WT

Traits	*d2014*	WT	Variation
Plant height (cm)	163.02 ± 2.00^***^	221.63 ± 2.17	−58.61
Ear height (cm)	35.97 ± 1.33^***^	80.56 ± 0.67	−44.59
Total tassel length (cm)	45.50 ± 5.67	47.37 ± 6.33	−1.87
Tassel branch number (cm)	11.67 ± 2.07	13.12 ± 4.00	− 1.45
Tasseling stage (day)	50 ± 0.32	50 ± 0.12	0
Total leaf number	20.49 ± 0.45	20.4 ± 0.38	0.09
Leaf number above the ear	7.06 ± 0.23	6.70 ± 0.27	0.36
Ear length (cm)	14.13 ± 0.49	15.04 ± 0.43	− 0.91
Ear width (cm)	4.59 ± 0.26	5.16 ± 0.20	− 0.57
Grain length (cm)	1.09 ± 0.02	1.12 ± 0.03	−0.03
Grain width (cm)	0.92 ± 0.01	0.89 ± 0.02	0.03
Ear weight (g)	122.12 ± 4.44	133.25 ± 4.89	−11.13
Hundred-grain weight (g)	32.35 ± 0.50	32.23 ± 0.43	0.12

Vaues indicate mean ± SD. *** represents significant difference between *d2014* and WT at 0.001 level

To ascertain the genetic basis of dwarfism for *d2014*, three F_2_ and three BC_1_ populations derived from *d2014* × HL5038, *d2014* × HL5054 and *d2014* × F19 were generated. F_2_ and BC_1_ populations presented tall or short individual separation. Through χ^2^ tests, the plant height segregated in a 3:1 ratio (tall/short) in all three F_2_ populations and in a 1:1 ratio (tall/short) in three BC_1_ populations (Additional file 2: Table S2). These results indicated that this dwarf phenotype of *d2014* was genetically controlled by a single recessive gene.

### The *d2014* gene is a *br2* allele

**Fig. 2:**
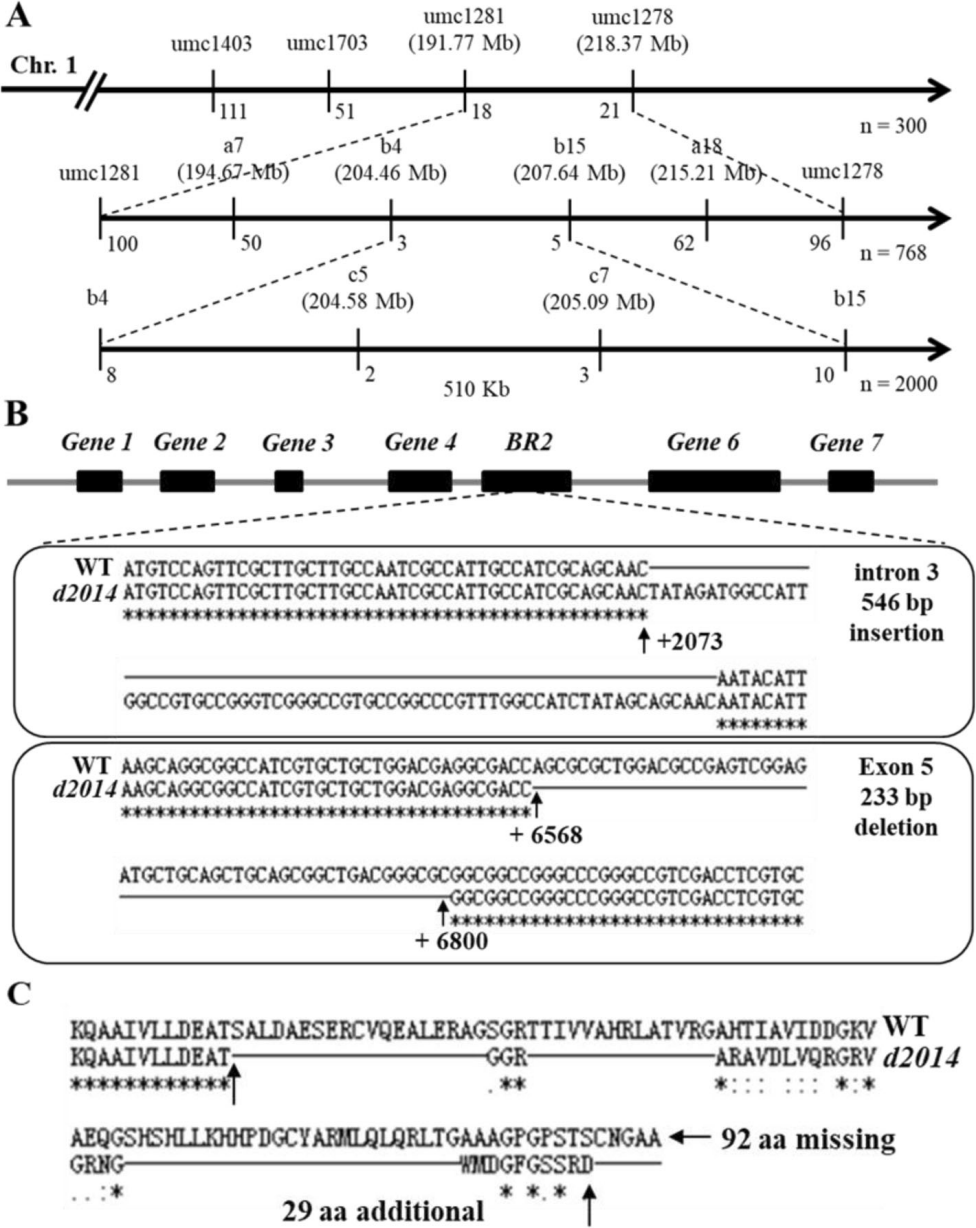
Identification of the *d2014* gene. **a** Positional cloning of the *d2014* gene: Firstly, the *d2014* locus was mapped between the markers umc1281 and umc1278 on the chromosome 1 using 300 dwarf individuals from (*d2014* × HL5054) × *d2014* BC1 population. Secondly, we enlarged the population and developed new Indel molecular markers. The location of the *d2014* gene was then narrowed down to a smaller segment flanked by the markers b4 and b15. Finally, using 2000 recessive plants, we locate the candidate region in about 470 Kb between the markers c5 and c7. The numbers below the vertical lines indicate the recombinants. **b** The *BR2* and other six predicted genes in the 470 Kb region, and and the sequence alignment of *BR2* in *d2014* and WT. **c** Variation of the BR2 protein sequence between *d2014* and WT

To clone the dwarf *d2014* gene, we carried out positional cloning using the (*d2014* × HL5054) × *d2014* BC_1_ population. Firstly, 300 BC_1_ individuals that had a similar dwarf phenotype as *d2014* were identified and genotyped by 150 pleomorphic simple sequence repeat (SSR) makers, and then the *d2014* gene locus was defined to a 40.05 Mb genetic interval between marker umc1281 and umc1278 (Fig. 2a). Subsequently, 768 dwarf plants were genotyped for fine mapping by newly developed InDel molecular makers, and the *d2014* gene was narrowed to a smaller segment flagged by the two markers a4 and a15, which are 2.85 Mb apart (Fig. 2a). Finally, 2000 recessive individuals were used to determine the candidate region of about 510 Kb that contained the *d2014* gene (Fig. 2a)*.*

Database analysis showed that *BR2* and six other candidate genes are within this 470 Kb region (http://ensembl.gramene.org/biomart/martview/; Fig. 2b). Genomic DNA sequences of these genes from the *d2014* mutant and WT were compared, and the results showed that only the *BR2* sequences in *d2014* had two variant regions, including a 546 bp insertion in intron 3 (C_2073_) and a 233 bp deletion (A_6568_ to C_6800_) in exon 5 (Fig. 2b). The inserted sequence in intron 3 seemed not to be characterized as transposable elements, while the missing region in exon 5 would result in the deletion of 92 amino acids and a frameshift affecting the translation of 29 additional amino acids (Fig. 2c). Moreover, a known *br2* mutant (114F) was crossed with *d2014* and WT, respectively. Significant differences in plant growth and development between the two hybrids were observed. At the 14-leaf stage, the morphological height of hybrid 114F × *d2014* was significantly lower than that of hybrid 114F × WT, and the internode length of the former was strongly shortened in comparison with hybrid 114F × WT (Additional file 3: Figure S1). Over all, these results confirmed that the *d2014* gene was a *br2* allele with a unique variation.

### The *d2014*/*br2* mutation alters the normal splicing of *BR2* gene

**Fig. 3:**
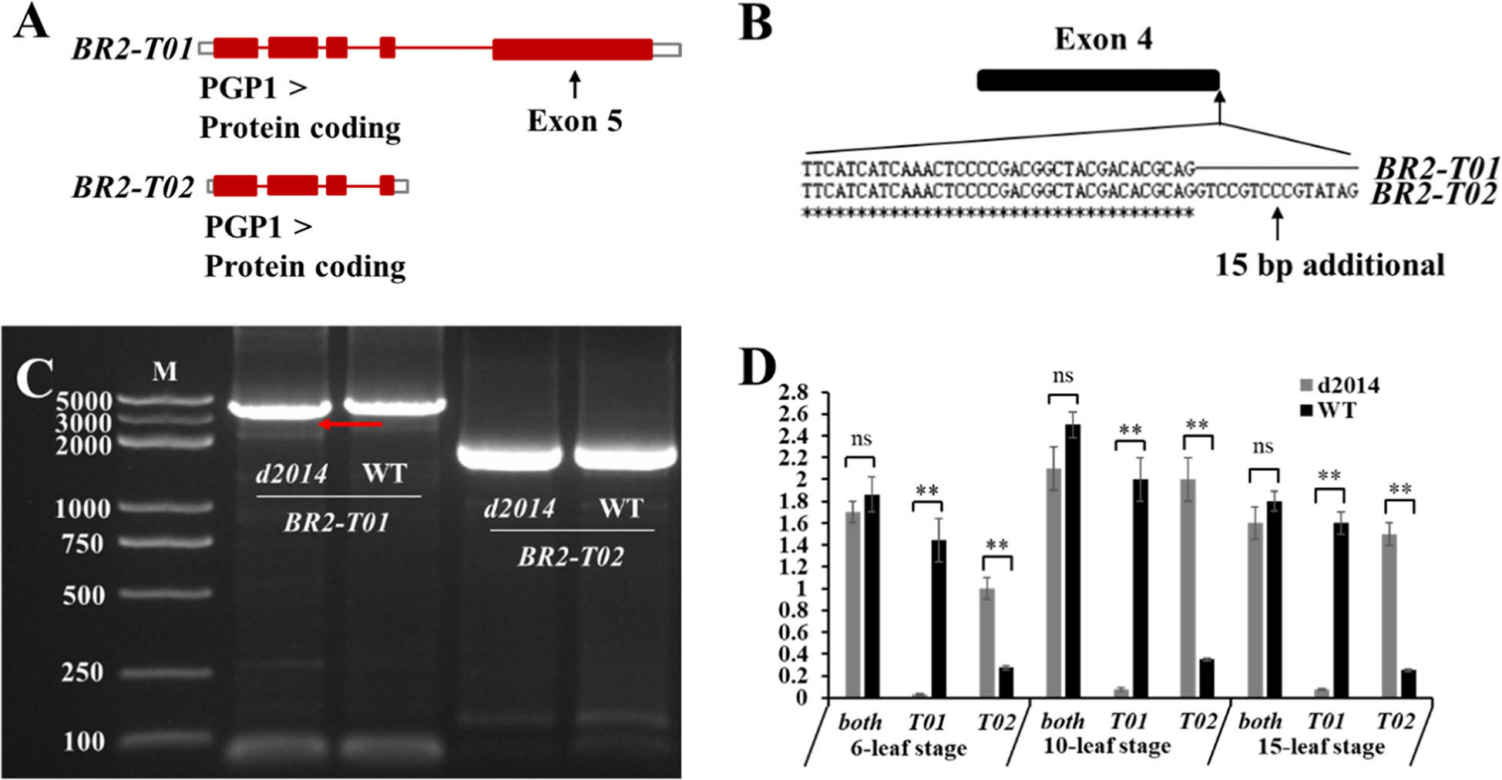
Identification of the two transcripts of *BR2*. **a** Distribution of the exons of *BR2-T01* and *BR2-T02*. **b** Differences in exon4 between *BR2-T01* and *BR2-T02*. **c** Molecular identification of *BR2-T01* and *BR2-T02*. The red arrow indicates the differences of the band size between mutant *d2014* and WT for *BR2-T01*. **d** The expression pattern of *BR2-T01* and *BR2-T02* in *d2014* and WT. ** *P* ≤ 0.01 (Student’s t-test); ns, not significant

Generally, a genetic mutation would cause the variation of natural expression pattern or protein function, resulting in phenotypic defects. Database analysis showed that *BR2* had two transcripts: *BR2-T01* (4672 bp) and *BR2-T02* (2061 bp) (https://www.maizegdb.org/). Actually, *BR2-T01* is composed of 5 exons, while *BR2-T02* only has 4 exons excluding the exon 5 (Fig. 3a). In addition to the 5’UTR, 3’UTR and exon 5, a vital region with an additional 15 bp in *BR2-T02* exon 4 is different from that in *BR2-T01* exon 4 (Fig. 3b). To verify the existence of these two transcripts in vivo, their cDNA sequences were amplified and compared. For *BR2-T01*, the band size of *d2014* was distinctly less than that of WT (Fig. 3c), which was in agreement with a fragment deletion in *d2014*. Sequencing the band revealed the previously predicted 233 bp deletion. Meanwhile, there was also a similar band of about 2000 bp for *BR2-T02* in *d2014* and WT (Fig. 3c), the sequences of which were identical. This suggested that the two transcripts truly existed in vivo.

In view of this, it was necessary to analyze the effect of *d2014*/*br2* mutation on the two transcripts. To define the expression patterns of *BR2-T01* and *BR2-T02*, qRT-PCR was carried out through specific quantitative primers. During the three stages of 6-leaf, 10-leaf and 15-leaf, the expression levels of *BR2-T01* in *d2014* were all lower than in WT, whereas *BR2-T02* was significantly up-regulated in *d2014* during the three stages (Fig. 3d). These data suggested that the natural expression of *BR2-T01* is crucial for WT normal growth, and the down-regulated *BR2-T01* leads to the defective traits of *d2014*. Interestingly, the total *BR2* expression level was roughly similar between *d2014* and WT (Fig. 3d). These results revealed that the *d2014* mutation alters the normal splicing of *BR2* gene, causing a higher expression of *BR2-T02* instead of normal *BR2-T01*.

### Analysis of BR2/PGP1 and auxin efflux in yeast

**Fig. 4:**
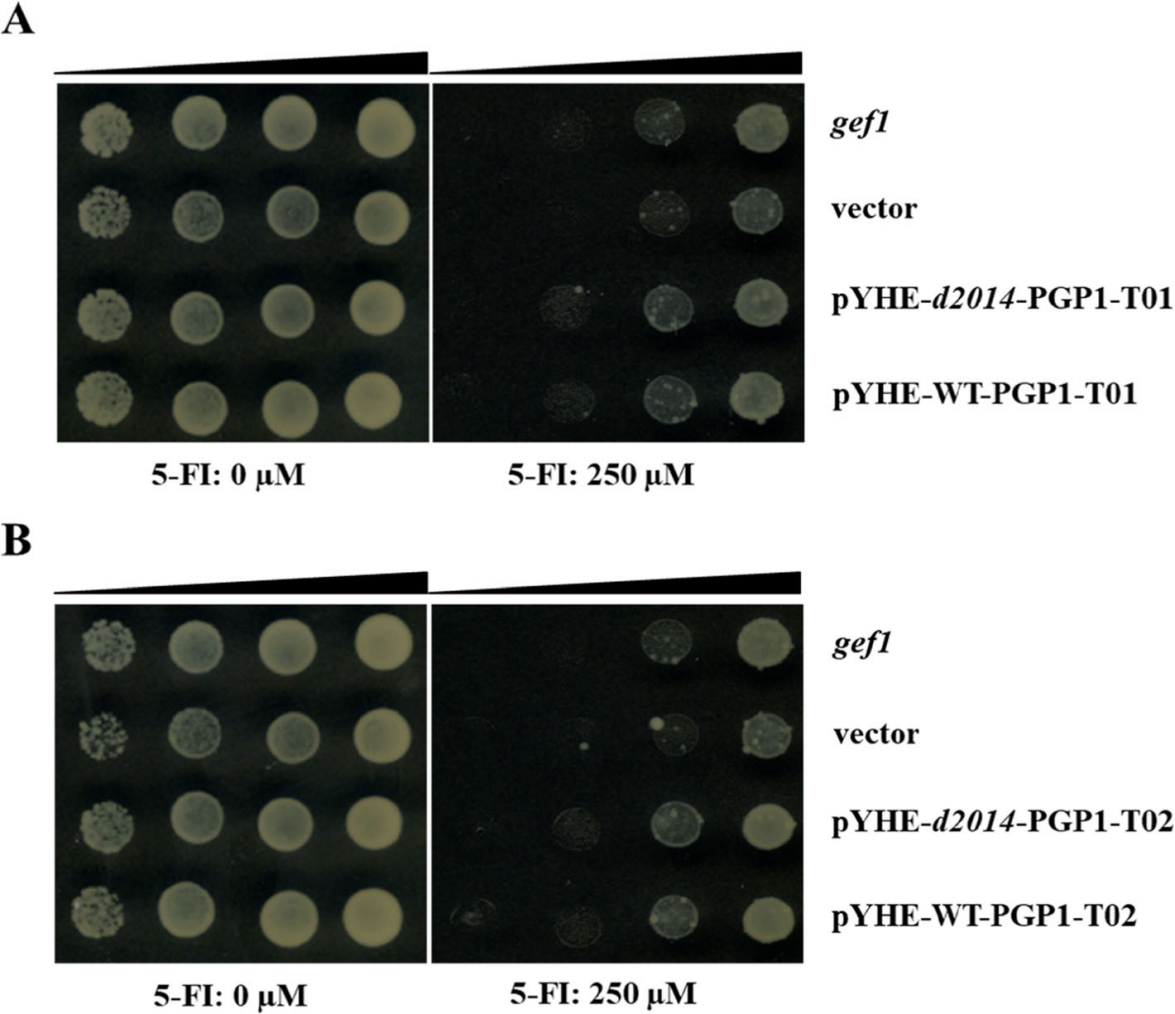
Functional analysis of protein PGP1-T01 and PGP1-T02. **a**, **b** Heterologous expression of PGP1-T01 and PGP1-T02 for detoxification assays in 5-FI hypersensitive *gef1* mutant strain, respectively. Represents that 1-, 10-, 100-, and 1000-fold dilutions (from right to left) of yeast cells were orderly spotted on YPDA medium containing 0 μM or 250 μM 5-FI, including the original strain *gef1*, the vector control and the transformed strains

In maize, *BR2* encodes an auxin transporter, PGP1, which functions in auxin export from intercalary meristems [34]. In order to investigate whether the protein BR2/PGP1 (T01 and/or T02) is responsible for auxin transport at the cellular level, we functionally expressed these two variants in yeast. Fluorinated indole derivate (5-FI), a toxic analog of indole-3-acetic acid (IAA, namely auxin), is cytotoxic to yeast and has been used to investigate auxin transport [35, 36]. Yeast mutant strain *gef1* lacks endogenous chloride channel protein [37], which results in 5-FI accumulation in *gef1* cytoplasm after the undissociated 5-FI molecules entering cells by passive diffusion, leading to *gef1*’s hypersensitivity to 5-FI. Using the yeast mutant strain *gef1*, we found that both of WT-PGP1-T01 and *d2014*-PGP1-T01 provided certain resistance against 5-FI compared to the vector control (Fig. 4a). The result indicated that the protein PGP1-T01 exported 5-FI to the outside of the yeast cell, namely, PGP1-T01 was responsible for auxin export. Interestingly, PGP1-T02 also exhibited slight but significant resistance against 5-FI in the yeast mutant strain *gef1* (Fig. 4b), indicating its functions in auxin export. In terms of alternative splicing, there are mainly two types: front mutation (T01 and T02, both defective) and back mutation (T01, defective; T02, normal). When the two proteins, PGP1-T01 and PGP1-T02, play similar roles in auxin’s export, the second type of mutation would display a mild phenotype, which opens the possibility that there is a variation of *br2* mutant with diverse defects.

### Defects of internode elongation in the *d2014* mutant

**Fig. 5:**
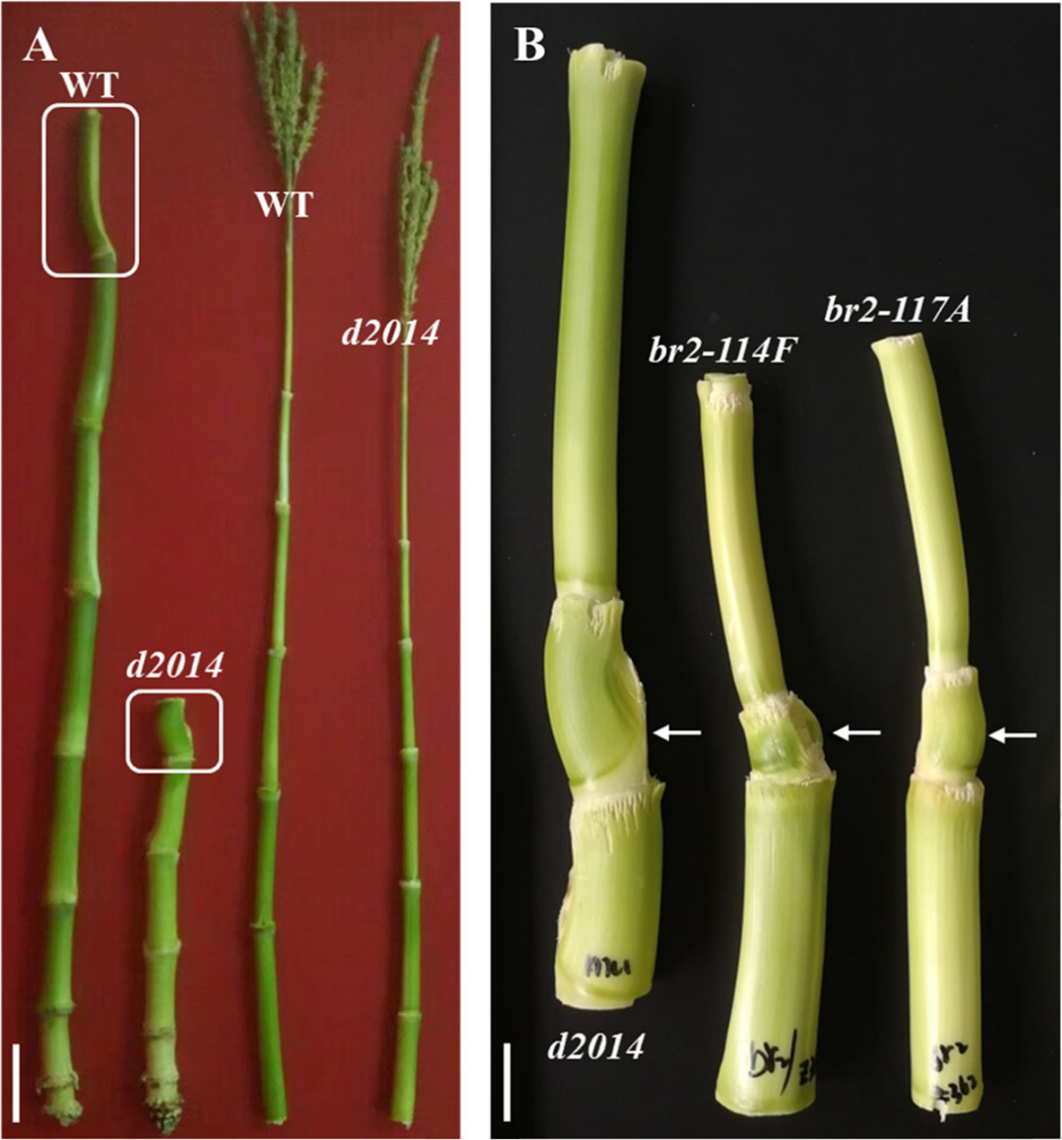
Morphological comparison of internodes at the mature stage. **a** Morphology of the internodes below the ear or above the ear between *d2014* and WT. The white box indicates ear-internode. *Bar* = 10 cm. **b** Morphology of the ear-internodes of the three *br2* mutants: *d2014*, *br2-114F*, and *br2-117A*. The arrows indicate the ear-internodes. *Bar* = 2 cm

A typical characteristic of *br2* mutants is that dwarf stature derives from shortened lower internodes [32, 34]. Of the *d2014*/*br2* mutant, the length of certain internodes below the ear was dramatically reduced; additionally, the ear-internode was also shortened, whereas the internodes above the ear were approximately normal (Fig. 5a). Meanwhile, we found that other *br2* mutants, *br2-114F* and *br2-117A*, also displayed an abnormal ear-internode phenotype similar to *d2014* (Fig. 5b). These data showed that the loss-of-function alleles of *br2* specifically regulated the elongation of certain internodes.

**Fig. 6:**
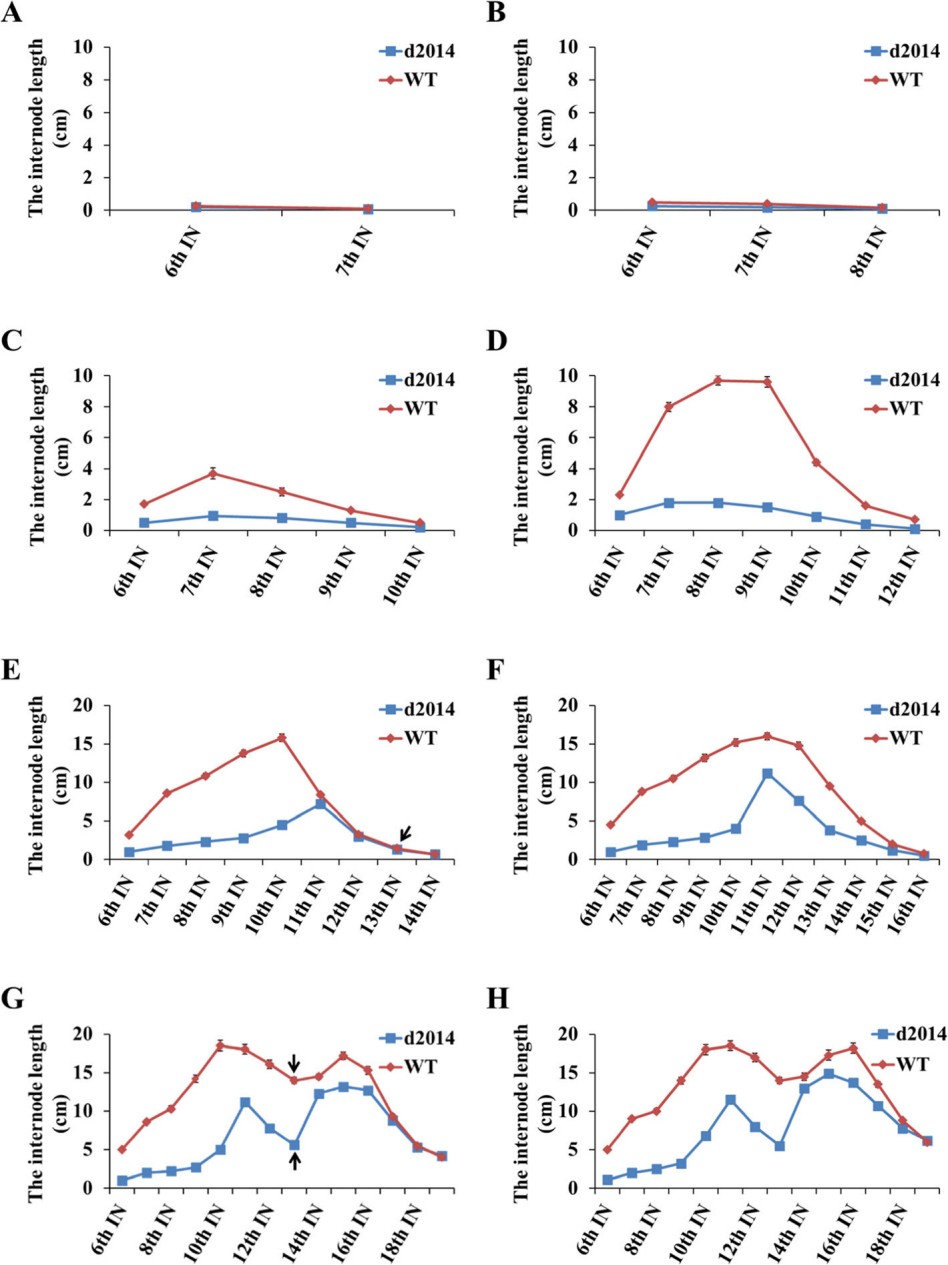
Dynamic observation of internode elongation. **a**, **b**, **c**, **d**, **e**, **f**, **g**, **h** The length of each internode at the 6-, 8-, 10-, 12-, 14-, 16-, 18-, 20-leaf stages, respectively. IN represents internode. At the 6-leaf stage, the first internode (6th internode) above ground is visible and then more internodes are elongated subsequently at other stages. The arrows in **e** and **g** indicate the ear-internode (13th internode)

Since the total leaf numbers and tasseling stage of WT and *d2014* (Table 1) are nearly the same, thus, their developmental phase should be paralleled. To clarify how the mutation of *br2* affects internode elongation, we carried out a dynamic comparison of stem growth between *d2014* and WT. Plenty of individuals were planted and leaf numbers were labeled to ensure the same developmental stage. Before the 12-leaf stage, the 6th, 7th, 8th, 9th, 10th, and 11th internodes in *d2014* were scarcely elongated (Fig. 6a, b, c, d), and the stem height of all these internodes was significantly lower than that in WT (Additional file 4: Figure S2A). From the 12-leaf stage to the 14-leaf stage, the11^th^, and 12th internodes in *d2014* grew rapidly compared to previous patterns, and their lengths increased (Fig. 6d, e). Moreover, the length of the 11th, 12th and 13th internodes (ear-internode) in *d2014* was similar to that of WT at the 14-leaf stage (Fig. 6e). Namely, the 5 lowest internodes in *d2014* were shortened seriously (Additional file 4: Figure S2B). Strangely, after the 14-leaf stage, the 11th, 12th and 13th internodes in *d2014* grew slowly in comparison with WT (Fig. 6e, f), and the elongation of the ear-internode was sharply restrained (Fig. 6g). Nevertheless, the patterns of upper internode elongation in both *d2014* and WT were exactly alike after the 16-leaf stage (Fig. 6g, h), which led to nearly paralleled length of the upper internodes (Additional file 4: Figure S2C). All these results revealed that defects of internode elongation in mutant *d2014* are disparate at different developmental stages; especially, at the 6-leaf, 12-leaf and 14-leaf stages, the internode elongation in *d2014* exhibits variation pattern of inhibited-normal-inhibited transiently.

### Effect of the *d2014* mutation on cell morphology in shortened internodes

**Fig. 7:**
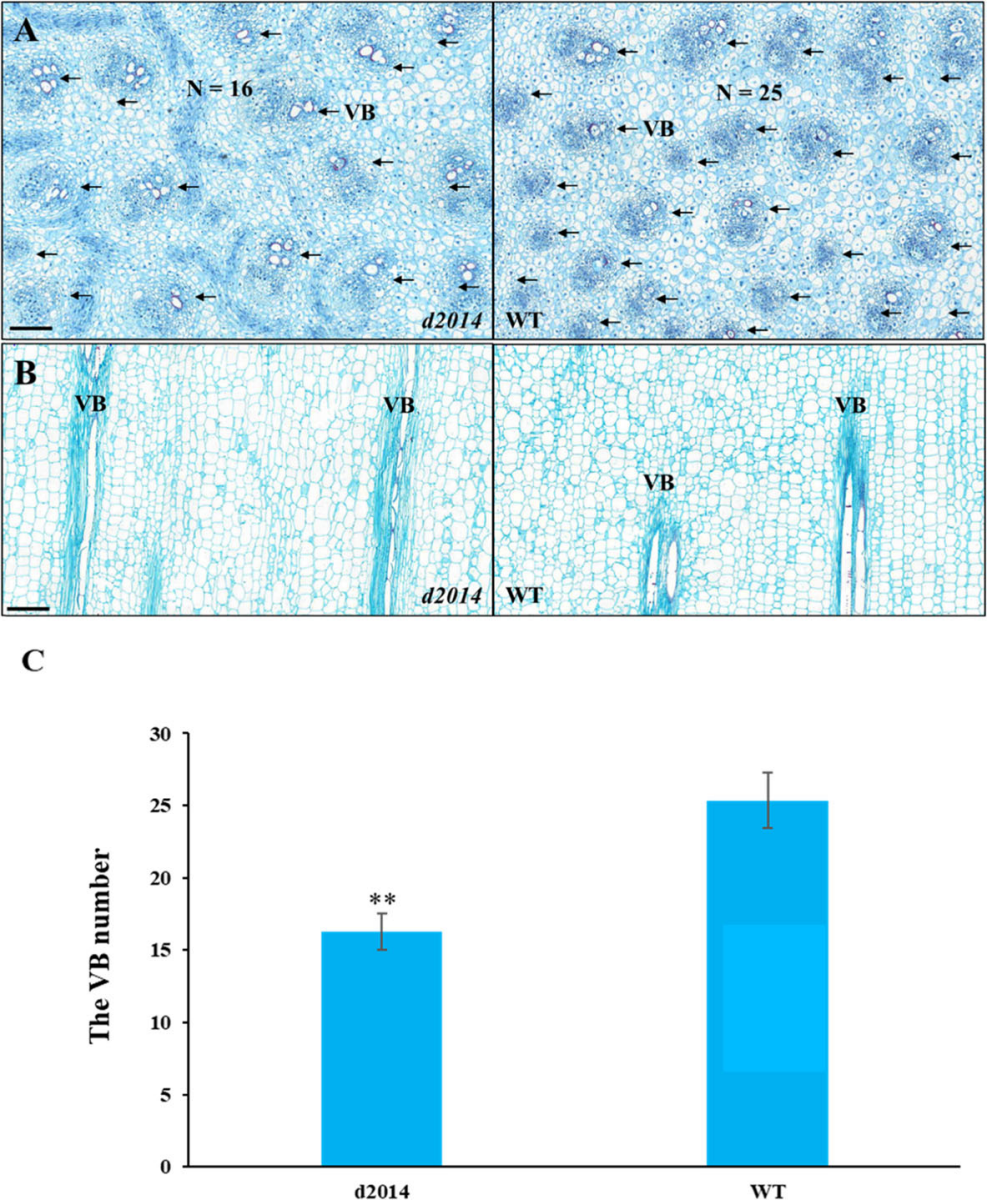
Morphological observation of the 6th internode cells of *d2014* and WT at the 6-leaf stage. **a** Cross-section of cells of *d2014* and WT. The arrows point to the vascular bundle (VB); N represents the total VB numbers. The magnification times are 20 ×. *Bar* = 150 μm. **b** Longitudinal section of cells of *d2014* and WT. The magnification times are 20 ×. *Bar* = 200 μm. **c** The statistics of VB number for the fixed center cross section. ** indicates the VB numbers in *d2014* were significantly reduced at *P* ≤ 0.01 level

At an early developmental stage (before 12-leaf stage), the elongation of the lower internodes in *d2014* was seriously inhibited. At the 6-leaf stage, the first over-ground internode (the 6th internode) was elongating. Though it seemed to be very short (Fig. 6a), there was a trending difference between WT and *d2014*. In order to understand the cell morphology of these shortened internodes in *d2014*, we observed the young 6th internode at the 6-leaf stage. Cross-sections showed that *d2014* had regular parenchyma cells similar to WT, and their cell sizes were comparable, whereas the numbers of vascular bundles in *d2014* had been significantly reduced (Fig. 7a, c). Longitudinal sections showed that the length of parenchyma cells was not changed between *d2014* and WT, but the interval of two adjacent vascular bundles in *d2014* was larger than that in WT (Fig. 7b), which meant that there was a reduced vascular bundle number for *d2014*. Thus, the *d2014* mutation originally affects the formation of vascular bundle in shortened internode cells.

### Location of *BR2* expression in the stem

**Fig. 8:**
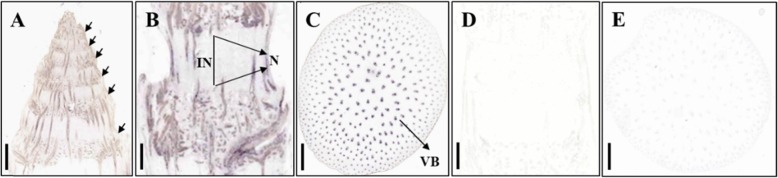
In situ localization of *BR2* mRNA in stem at the 10-leaf stage. **a** Longitudinal section of stem apex. The arrows represent each node in stem apex. *Bar* = 0.1 cm. **b** Longitudinal section of an intact internode. IN, internode; N, node. *Bar* = 0.3 cm. **c** Transverse section of longest internode. VB, vascular bundle. *Bar* = 0.2 cm. **d**, **e** Hybridization of a single internode with a *BR2* sense probe for longitudinal section and transverse section, respectively. *Bar* = 0.2 cm

Since the mutant *d2014*/*br2* displays defects in internode elongation, we predicted that *BR2* is expressed in these tissues. However, the expression pattern of *BR2* has not been precisely defined previously. To figure out the location of *BR2* expression in the stem, we performed in situ RNA hybridization at the 10-leaf stage. In longitudinal sections of the stem apex, *BR2* expression was clearly localized in each node (Fig. 8a). The upper and lower nodes revealed clear signals in the single internode on the ear (Fig. 8b). In cross sections of the longest internode, *BR2* mRNAs were observed in a mass of vascular bundles (Fig. 8c). A single internode was selected for control experiments, in which the sense probe produced no signal (Fig. 8d, e). In maize stems, the vascular bundles interlace in nodes and then tilt into internodes with multiple branches, which finally form a network pipe from top to bottom. Thus, *BR2* expression was mainly localized in vascular bundles of the node and internode. Xing indicated that the subcellular localization of QPH1 (BR2) is exclusively in membrane [33]. There is no variation in the signal peptide region of *d2014*/*br2*, thus, mutation should not influence the subcellular localization of *d2014*/*br2*. In WT, the expression level, localization and transport activity of BR2 are normal, relatively; while under other circumstances (for example *br2* mutation), developmental defects occur. Overall, these data indicated that PGP1 functions in auxin export from vascular bundles in stem, then regulating internode elongation.

### Quantitative analysis of auxin in the *d2014* stem

**Fig. 9:**
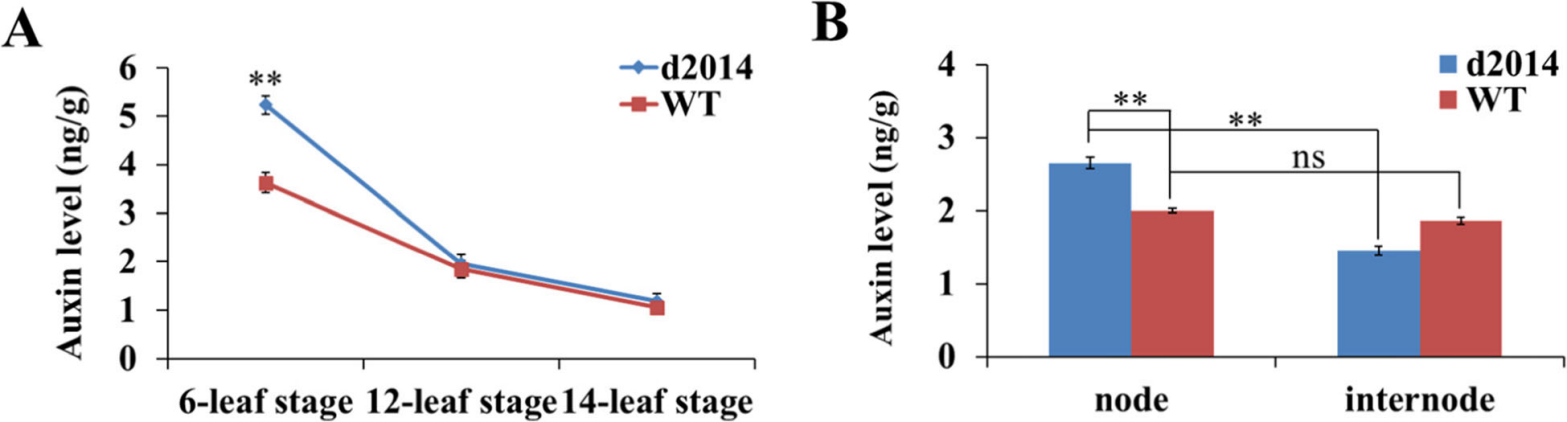
The change of auxin concentrations in the stem at different stages. **a** Auxin levels of stem apex in *d2014* and WT at the 6-, 12-, and 14-leaf stages. **b** Auxin levels of a single ear-internode including node and internode region at the 14-leaf stage. ** indicates significant difference at 0.01 level by Student’s t-test. ns, not significant

Auxin transport influences the distribution and local concentration of auxin [38]. In the prior studies, *br2* mutation influences auxin transport and local concentration, which are responsible for the dwarf phenotype [32–34]. The defective auxin transport in *d2014* stems, mediated by BR2/PGP1, would cause the auxin concentration variation in the stem and then influence the internode elongation. To investigate the links between the two, we detected the auxin concentration in several key periods of internode elongation variation (as mentioned above). At the 6-leaf stage, auxin concentration in the *d2014* stem apex was significantly higher than that in WT (Fig. 9a). We also measured the auxin concentration in the stem apex of *d2014* and WT at the 12-leaf and 14-leaf stages. However, auxin concentration was gradually reduced compared with the former period (Fig. 9a). Moreover, little difference was observed between *d2014* and WT at these two stages (Fig. 9a). These showed that the *d2014*/*br2* caused higher auxin level in the stem apex at the early development stages, at least at the 6-leaf stage, and correspondingly, the internode elongation of *d2014* was suppressed. In addition, we examined the auxin levels of the single ear-internode at the 14-leaf stage, including two parts: the node and the internode region. Interestingly, the auxin level of the nodes in *d2014* was significantly higher than the nodes in WT, as well as the internode region itself (Fig. 9b), while in WT, there was no significant difference (Fig. 9b). Furthermore, the auxin levels of the two parts exceeded the stem apex at the 14-leaf stage (Fig. 9a; b). These data revealed that auxin at the 14-leaf stage was increased for ear-internode and was detained in the node region of *d2014*. Notably, the ear-internode in *d2014* began to grow rather slowly after the 14-leaf stage and finally was extremely shortened. Taken together, these data showed that the *d2014/br2* caused increasing in auxin levels in the shortened lower internodes (stem apex at the 6-leaf stage) and ear internodes (node region at the 14-leaf stage), which were responsible for the defects of internode elongation.

## Discussion

### *d2014* gene, with a splicing variation, is a potentially useful *br2* allele

In maize, there have been many reports on the identification of *br2* mutants, which involved a variety of the mutation loci (Additional file 5: Figure S3). At first, the *BR2* gene were cloned and confirmed by transposon tagging with *Mutator* (*Mu*), such as *br2–6*, *br2–7* and *br2–9* with *Mu* insertion in exon 1, as well as *br2–3* with *Mu* insertion in intron 4 [32]. Subsequently, several other *br2* alleles, such as one SNP variant (G/T5295) in exon 5 [33] and a 241 bp deletion (G6367 to C6617) in exon 5 [39], were identified to result in the partial loss of BR2/PGP1 protein function. All the above *br2* mutants were derived from multiple inbred lines with a different genetic background. Nevertheless, they all presented shorter lower internodes, but nearly normal upper internodes. Without exception, our *d2014br2* displayed similar features, which were verified by allelism test with *br2-114F*. Apart from the above phenotypes, we also found that the *br2* mutants (*d2014*, *br2-114F* and *br2-117A*) displayed a shortened ear-internode. Therefore, it is certain that this *br2* allele is responsible for the shortened internodes.

In the maize B73 reference genome, *BR2* has two predicted splicing variants: *BR2-T01* and *BR2-T02*. In this study, we identified a new *br2* allele (*d2014*), which contained a 546 bp insertion in *BR2* intron 3 (C_2073_) and a 233 bp deletion (A_6568_ to C_6800_) in *BR2* exon 5. Sequencing of the cDNA confirmed the presence of the two *BR2* splicing variants, and *BR2-T02* was highly expressed in *d2014* while *BR2-T01* expression level was very low, which was contrary to that in WT. Similarly, the two *BR2* splicing variants have been verified molecularly in a dwarf *br2-NC238* allele mutant [40]. The insertion of a novel transposon in *BR2* intron 4 alters the normal splicing of the gene, which results in abundant expression of *BR2-T02* in *br2-NC238* instead of *BR2-T01* in tall NC238 plants. Generally, insertion, deletion, or single-base replacement of the exon region may result in the defective protein with altered function; while the variation of the intron region might affect gene expression pattern through changing the mRNA splicing. Therefore, we concluded that the insertion of 546 bp in intron 3 altered the normal splicing of *BR2* gene, which was the main reason for the dwarfing of *d2014*. Overall, *d2014* with a splicing variants has the enriched types of *br2* allele.

Some studies have shown that some different splicing variants perform equivalent functions to the constitutive form, even when lacking amino acid sequences or protein domains [41]. The *BR2* gene encodes an auxin transport protein, PGP1, which is an ATP-binding cassette (ABC) transporter [42]. In general, full-size ABC transporters consist of two similar halves, each containing a transmembrane domain (TMD) and a nucleotide binding domain (NBD) [43]. The so-called half-size ABC transporters only have a half (one TMD and one NBD) and require dimerization for their transport activity [43]. An example is ECERIFERUM5, which is a half-size ABC protein and functions in wax export to the plant cuticle [44]. Here, through the analysis of protein domains by (http://smart.embl-heidelberg.de/smart), we found that PGP1-T01 was a full-size ABC transporter, while PGP1-T02 contained exactly a half, making it a half-size ABC transporter (Additional file 6: Figure S4). Diana Santelia and Markus Geisler examined the auxin transport properties (influx or efflux) of ABCB protein by heterogeneous expression in yeast [35, 36]. Since the strong promoter was used to induce the overexpression of ABCB protein, it would not accurately evaluate the auxin transport activity. Similarly, our heterogeneous expression assays using strong promoter identified the transport properties of the two proteins PGP1-T01/T02, which indicated that PGP1-T01 (even including the defective d2014-PGP1-T01) and PGP1-T02 have played similar roles in auxin efflux. Previously, Multani and Knöller have confirmed that *br2* mutation leads to the decrease of auxin flow from top to bottom, which is responsible for the dwarf phenotype [32, 34]. In our study, the expression of *br2-T01* in *d2014* was significantly lower than that of *BR2-T01* in WT (normal status), indicating that the normal auxin transport activity in *d2014* was impaired. Nevertheless, our phenotypic analysis showed that *d2014* was a mild dwarf mutant with nearly no other undesirable traits, which should be attributable to the compensation of functional PGP1-T02. Actually, in the F_2_ population of *br2-qph1* and *br2-117A*, plant height and ear height segregated in a 3:1 ratio (*br2-qph1*/− plants to *br2-117A*/*br2-117A* plants), which indicated that *br2-qph1* was dominant to *br2-117A* [33]. Namely, different *br2* alleles would give rise to certain defects in varying degrees. Therefore, our work shows a useful *br2* allele that has beneficial potential for maize improvement by moderately reducing the plant height while not affecting the yield.

### BR2/PGP1 functions in auxin efflux from the vascular bundles of the node and internode

Auxin is often synthesized in the apex region and is directionally transported to the lower region [45, 46]. This directional auxin flow is attributable to membranous auxin transporters. So far, three major families of auxin transporters have been identified: AUXIN-RESISTANT1 (AUX1)/AUX1-LIKEs (LAXs) for auxin influx and PINFORMEDs (PINs) and several PGP proteins for auxin efflux [47]. Some studies indicated that ABCB efflux transporters might limit auxin reuptake at efflux sites for assisting PIN-oriented auxin flow [48]. In addition, several studies suggested that ABCBs may play a role in local auxin loading of long-distance auxin transport [49, 50].

BR2/PGP1 is an orthologue of *Arabidopsis thaliana* ABCB1/PGP1, which is expressed in shoot and root apices and functions primarily in auxin efflux from meristematic cells into long-range auxin transport streams [34, 51, 52]. Previously, Knöller applied [^3^H]-IAA to the upper stem of maize (B73 vs *br2*), and found that several [^3^H]-IAA accumulation peaks were detected near the nodes; meanwhile, the moving front was reduced and free auxin levels in the node of *br2* mutant are significantly higher than in adjacent internodes [34]. Consistently, in our *d2014*/*br2*, higher auxin levels were detected in the stem apex (including multiple nodes) at the 6-leaf stage and in the node region of the ear-internode at the 14-leaf stage. In a word, the *br2* mutation caused auxin accumulation in the node regions. Notably, our in situ hybridization assay showed that *BR2* is primarily expressed in vascular bundles (VBs) of the nodes and internodes. In line with this, Anne Sophie Knoller indicated that BR2 is mainly expressed in the nodal region (dense VBs) [34]. In this context, we propose that BR2/PGP1 functions in auxin efflux from the node to the internode through vascular bundles. Thus, auxin efflux is reduced from the nodes to the internodes in *br2* mutant, and auxin level is higher in *br2* node regions.

### Excessive auxin levels in the intercalary meristem region suppress internode elongation

Internode elongation is controlled by several factors, mainly the plant hormones. In this study, we found that the variation of auxin level in *d2014* stem influences internode elongation. At the 6-leaf stage, the elongation of the lower internodes began to be severely suppressed in *d2014*, along with higher auxin levels in the stem apex compared with WT. As the auxin levels of the stem apex gradually decreased at the 12-leaf and 14-leaf stages, internodes in *d2014* grew rapidly. At the 14-leaf stage, additionally, the auxin level of the node region next to the ear in *d2014* was much more than that in WT, corresponding to the irregular and shortened ear-internode. In short, *d2014*/*br2* and WT have the same genetic background except the *br2* locus, causing the shortened internodes, as well as higher auxin levels in their node regions, which suggested that higher auxin levels in *d2014* stems (node regions) suppress the internode elongation.

Generally, auxin has fundamental roles in rapid stimulation of cell expansion for promoting growth [53, 54]. This seems to be contradictory with what we found in this study. Nevertheless, similar scene that auxin inhibits growth is fit for apical dominance in plant. Growing shoot apexes produce an inhibitory hormone, auxin, which moves downwards within the stem and inhibits the growth of axillary buds (including the AM) next to the node [55, 56]. Recent studies have revealed that a local auxin minimum is critical for AM formation or initiation [57–59]. Correspondingly, the stem of some plants, including maize, contains intercalary meristems that support stem growth independently of the shoot apex [5, 60]. Intercalary meristems are located in the internodes immediately above a node, by which the internode can elongate [61]. Moreover, our cytological analysis showed that the *d2014* mutation causes a reduced number of vascular bundles, which should be related to intercalary meristem functions. In concordance with this, Multani has also observed the cellular architecture of the *br2* internodes, and their data showed that the VB numbers of 1th over-ground internode (6-week-old) are reduced [32]; at the same time, Xing found that the VB numbers of 6th over-ground internode (19-leaf stage) are altered (reduced without specific statistics) in *br2* mutants [33], which indicated that less-elongated internodes in *br2* have reduced VB numbers. Some studies have shown that cell division or differentiation dependent on auxin transport flow is crucial for the formation of vascular bundles in the leaf [62]. Therefore, we speculate that a low auxin level is indispensable for intercalary meristem functions and, in reverse, internode elongation is suppressed when excessive auxin is arrested in intercalary meristem region.

**Fig. 10:**
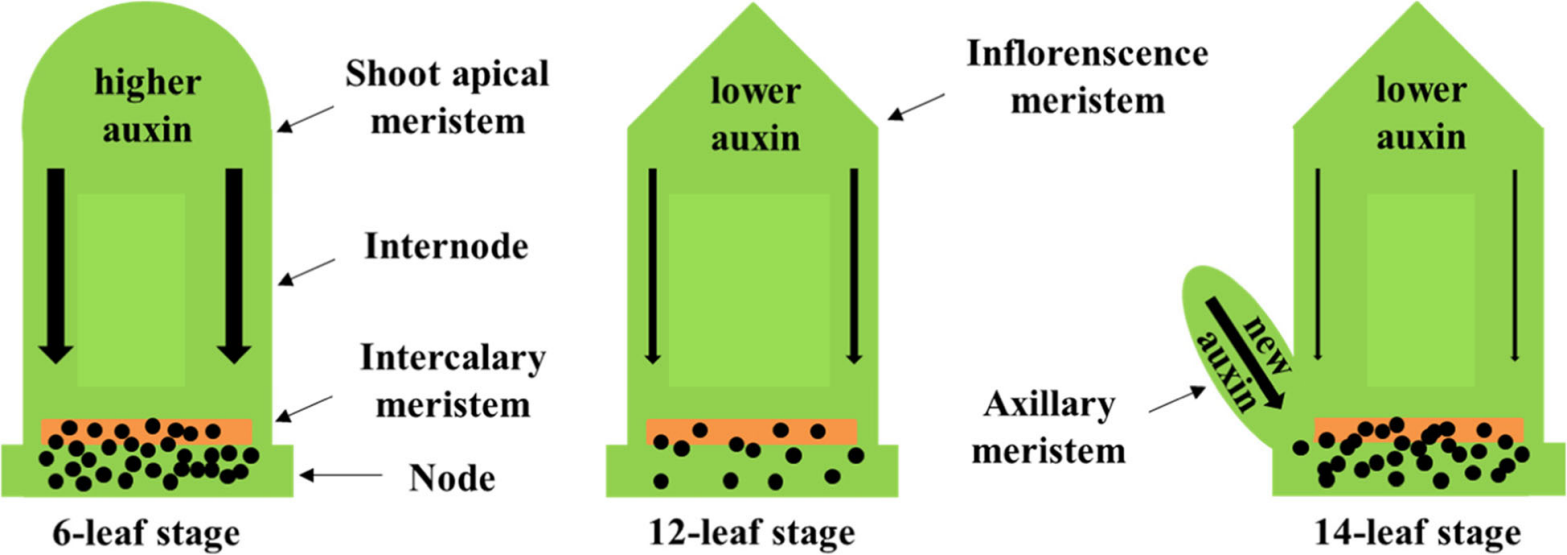
The sketch map of auxin flow in stem of *d2014* mutant during the internode development. The bold arrow represents the direction of auxin flow and the thickness represents the magnitude of auxin flow

All the *br2* mutants presented shortened lower internodes, as well as ear-internode, but nearly normal upper internodes. Through dynamic observation of internode elongation, we found that the variation of internode elongation in *d2014* is closely related to the 6-leaf, 12-leaf and 14-leaf stages. Notably, at the 6-leaf stage, the first internode (6th internode) above the ground was visible, and more internodes elongated subsequently (Additional file 7: Figure S5A); whereas, the juvenile tassel came into view at the 12-leaf stage (Additional file 7: Figure S5B); in addition, the ear-internode, at the 14-leaf stage, was undetectable until the upmost AM emerged (Additional file 7: Figure S5C). These three stages involve the growth and development of shoot apical meristem (SAM), inflorescence meristem (IM) and AM, which is associated with the synthesis of auxin. In context, we present a model to illuminate how loss-of-function of *br2* alleles uniquely regulates internode elongation (Fig. 10). At early stages, vigorous auxin is synthesized in the SAM region, and then excessive auxin is arrested in the intercalary meristem region of the lower internodes, and finally, elongation of the lower internodes is suppressed. Along with SAM translating to IM, declining auxin is generated in the apex region, and the upper internodes grow normally. For the severely shortened ear-internode, the chief culprit is a new auxin source derived from the developmental AM, which promotes abundant auxin being restricted in the intercalary meristem region of the ear-internode. Overall, the BR2/PGP1-dependent low auxin level in the intercalary meristem region is crucial for internode elongation.

## Conclusions

Plant height is one of most important agronomic traits in crop breeding. Maize BR2/PGP1 regulates the plant height by promoting auxin efflux from node to internode and avoiding excessive auxin accumulation in the intercalary meristem region to suppress the internode elongation. These findings are of great significance to the decryption of genetic mechanism of plant height and the improvement of maize yield.

## Methods

### Plant materials

The *d2014* is a dwarf mutant derived from the HL9047 inbred line (as WT), which was bred by our pedigree method after 9-generation selection. Three different tall stature inbred lines (HL5038, HL5054 and F19) were selected from our high generation breeding materials to construct F_2_ and BC_1_ populations with *d2014*. In addition, the *br2* mutants were requested from the Maize Genetic Cooperation Stock Center (stock number: 114F and 117A), the *br2-114F* and *br2-117A* were identified and amplified by our group for *br2* allelism test. All the plants were permissively cultivated in the standard experimental field of Sichuan Agricultural University in Sichuan during the summer and in Yunnan during the winter.

### Positional cloning of the *d2014* gene

The BC_1_ population (HL5054 × *d2014*) × *d2014* was used, and 6000 individuals were prepared to define the *d2014* gene locus. Indeed, using the simple sequence repeat (SSR) makers obtained from the MaizeGDB Database and Indel molecular makers acquired from Liu Jian research group in Sichuan Agricultural University, *d2014* gene was mapped to chromosome 1 bin 1.06, a ~ 510 Kb region with *BR2* and six other predicted genes. To identify the mutation site(s) of *d2014* gene, we amplified the DNA sequences of these genes and compared them between *d2014* and WT. Meanwhile, we used the *br2* mutant 114F to examine the allelism between *d2014* gene and *BR2.*

### RNA isolation and quantitative real-time PCR (qRT-PCR) analysis

At the 6-leaf, 10-leaf and 15-leaf stages, stem apexes (about 0.5 cm) in *d2014* and WT were flash-frozen by liquid nitrogen and stored at − 70 °C. Thereinto, the stem apexes are the top internodes except the juvenile tassel at the 10-leaf and 15-leaf stages. Total RNA was extracted using Trizol reagent, and the quantity and purity of RNA was determined by a NanoDrop 2000 and 1% agarose gel electrophoresis. Then, RNA was reverse-transcribed to cDNA using the PrimeScript™ RT-PCR Kit with gDNA Eraser (TaKaRa). The database showed that *BR2* has two splicing forms corresponding to two transcripts: *BR2-T01* and *BR2-T02* (https://www.maizegdb.org/). PCR amplification of these CDSs was performed, and the PCR products were detected by electrophoresis and then sequenced. In addition, qRT-PCR was performed in a Bio-Rad CFX96 Real-Time PCR System with SYBR Green PCR Master Mix (TaKaRa). Specific primers were designed for distinguishing *BR2-T01* and *BR2-T02*. The primer Q-*BR2-T01* was designed to span exons 4 and 5, while the primer Q-*BR2-T02* covered exon 3, exon 4, and the 3’UTR region of *BR2-T02*. In addition, we designed the primer Q-*BR2-both*, which covered exons 3 and 4, for detecting the total *BR2* expression level. After normalization to *ACTIN1* expression as an internal control, the relative expression levels were measured through the 2^–ΔΔCt^ method. All primer sequences are listed in Additional file 1: Table S1.

### Heterogeneous expression assays in yeast

The yeast mutant strain *gef1*, which is hypersensitive to the IAA toxic analog 5-Fluoroindole (5-FI), was used to explore auxin transport properties [35, 36]. Yeast *gef1*, ATCC®4036838™, was ordered from the American Type Culture Collection (ATCC). Firstly, yeast shuttle vector pGADT7, which has two *Hind*III restriction enzyme cutting sites, was digested by *Hind*III to remove the nuclear localization signal (NLS) sequence. In addition, the expression of the transcript on vector pGADT7 is driven by a strong promoter P_*ADH1*_, so the yeast transgenics are similar to the overexpressed lines. Then, the CDSs of *BR2-T01* and *BR2-T02* were acquired and inserted into pGADT7-*Hind*III by homologous recombination (single- or multi-fragment), generating pYHE-*d2014*-PGP1-T01, pYHE-*d2014*-PGP1-T02, pYHE-WT-PGP1-T01 and pYHE-WT-PGP1-T02. All the recombinant vectors were introduced into the yeast mutant strain *gef1*. For leucine-deficient type *gef1*, single colonies were screened in synthetic minimal medium without leucine (SD-Leu^−^). Lastly, transformants grown in SD-Leu^−^ to OD_600_ = 0.8 were washed and resuspended in water to OD_600_ = 1.0. Cells were 10-fold, 100-fold, 1000-fold diluted, and 2.5 μl of each was spotted on YPDA medium containing 0 μM or 250 μM 5-FI [36] with the corresponding vector control and the yeast mutant strain itself as controls. Pictures were taken after 3–6 days of growth at 28 °C. All primer sequences in homologous recombinant are listed in Additional file 1: Table S1.

### Dynamic observation of internode elongation

Each 700 *d2014* and WT individuals were planted in a specially designed plot. The plot consisted of 3-m-long rows separated by 1 m space between each row. Five-row interval planting was performed for *d2014* and WT with 10 replicates, and 14 individuals were planted in each row. The 5th leaf and 10th leaf of uniform individuals were labelled in the field. In general, the first internode above the ground is visible when the 6th leaf is unfolded at the 6-leaf stage. Thus, we measured each visible internode length from the 6-leaf stage to the maturation stage, including the 6, 8, 10, 12, 14, 16, 18, and 20-leaf stages. At each stage, 10 leaf-labelled individuals in adjacent rows were investigated, and the average value was the phenotypic value of internode corresponding to the leaf number.

### Histocytological analysis

A plenty of WT and mutant individuals were planted (as described above). At the 6-leaf stage, the first over-ground internode (the 6th internode) elongated. We randomly picked three individuals of WT and mutant, respectively, as three biological replicates for cytological observation of the internodes. The whole 6th internode was cut except for two terminals (the node regions) to make a paraffin section. All samples were vacuumed quickly and fixed in FAA composed of 5% of formaldehyde (40% v/v), 5% of acetate, and 90% of 75% alcohol (v/v) overnight. The paraffin sections were made as described previously [67]. Firstly, the transection was made from the middle part (about 0.1 cm from the upper part). Next, the longitudinal section was made from the rest of the upper part. The cell morphology was observed through an optical microscopy. Based on the same area (the middle region) and the same magnification (20 ×), we counted all the vascular bundles (VBs) for the transection.

### In situ hybridization analysis

At the 10-leaf stage, tissues of WT were prepared, including the stem apex (about 0.5 cm segment from the tip), longest internode and single internode (about 1 cm). In situ hybridization for *BR2* was performed as described previously [63]. An antisense RNA probe labeled by the Digoxin marker was uniquely designed in the *BR2* exon 1 region, and sequences are listed in Additional file 1: Table S1. To reduce the disturbance of the background signal, pilot experiment was conducted to optimize the probe dosage. In addition, a single internode was selected for the control test with a sense probe (Additional file 1: Table S1).

### Endogenous auxin concentration analysis

The stem apex segment (about 0.5 cm) was prepared in several periods, including 6-, 12-, and 14-leaf stages. All tissues were flash-frozen by liquid nitrogen and stored at − 70 °C. The measurements of endogenous auxin were performed through liquid chromatography tandem mass spectrometry (LC-MS). Three repeats with three plants in each replicate were performed. Furthermore, at the 14-leaf stage, the ear-internode was divided into two parts, including the basal node segment (about 0.2 cm) and the middle internode segment (about 0.2 cm), the auxin concentration of which was also measured as above.

## Supplementary information

**Additional file 1: Table S1.** Probe sequences for *BR2* in situ hybridization and primers used for sequencing and qRT-PCR as well as homologous recombinant.

**Additional file 2: Table S2.** Plant height separation performance in F_2_ and BC_1_ populations.

**Additional file 3: Figure S1.** The *br2* allelism test for *d2014*.

**Additional file 4: Figure S2.** Comparison of certain internode length between *d2014* and WT at the 12-leaf, 14-leaf, and 20-leaf stages.

**Additional file 5: Figure S3.** Different mutation sites of *br2* alleles.

**Additional file 6: Figure S4.** The analysis of protein domains of PGP1-T01 and PGP1-T02.

**Additional file 7: Figure S5.** The morphological characteristics of the stems of *d2014* and WT.

## Data Availability

All data generated or analyzed during this study are included in this published article and its additional files.

## Abbreviations

5-FI: 5-Fluoroindole
ABC: ATP-binding cassette
AMs: Axillary meristems
*an1*: *Anther ear1*
ATCC: American Type Culture Collection
AUX1: AUXIN-RESISTANT1
*br2*: *Brachytic2*
BRs: Brassinosteroids
*d1*: *Dwarf1*
*d2014*: *Dwarf2014*
*dw3*: *Dwarf3*
GAs: Gibberellins
IM: Inflorescence meristem
LAXs: AUX1-LIKEs
LC-MS: Liquid chromatography tandem mass spectrometry
*Mu*: *Mutator*
*na2*: *Nana plant2*
NBD: Nucleotide binding domain
NLS: Nuclear localization signal
PGP1: P-glycoprotein1
PINs: PINFORMEDs
qRT-PCR: Quantitative real-time PCR
SAM: Shoot apical meristem
SLs: Strigolactones
SSR: Simple sequence repeat
TMD: Transmembrane domain
VBs: Vascular bundles
*vt2*: *Vanishing tassel2*
WT: Wild type
