# Supplementary material for: Maize brachytic2 (br2) suppresses the elongation of lower internodes for excessive auxin accumulation in the intercalary meristem region
Source: BMC Plant Biol. 2019 Dec 27;19:589. doi: 10.1186/s12870-019-2200-5 (PMC6935237; doi:10.1186/s12870-019-2200-5)
Supplement: Supplementary file 2 — Additional file 2: Table S2. Plant height separation performance in F2 and BC1 populations. [file 12870_2019_2200_MOESM2_ESM.docx]

**Table S2** Plant height separation performance in F_2_ and BC_1_ populations

| Populations | Plant phenotype | | | | |
| --- | --- | --- | --- | --- | --- |
|  | tall | short | Segregation ratio | χ^2^ | χ^2^_0.05_ |
| （F19 × *d2014*） F2 | 604 | 182 | 3.31 : 1 | 1.43 | 3.84 |
| （HL5054 × *d2014*）F2 | 706 | 242 | 2.92 : 1 | 0.14 |  |
| （HL5038 × *d2014*）F2 | 671 | 215 | 3.12 : 1 | 0.25 |  |
| （F19 × *d2014*） BC1 | 457 | 443 | 1.03 : 1 | 0.22 |  |
| （HL5054 × *d2014*）BC1 | 469 | 447 | 1.05 : 1 | 0.53 |  |
| （HL5038 × *d2014*）BC1 | 376 | 336 | 1.12 : 1 | 2.25 |  |
