# Supplementary material for: Maize brachytic2 (br2) suppresses the elongation of lower internodes for excessive auxin accumulation in the intercalary meristem region
Source: BMC Plant Biol. 2019 Dec 27;19:589. doi: 10.1186/s12870-019-2200-5 (PMC6935237; doi:10.1186/s12870-019-2200-5)
Supplement: Supplementary file 3 — Additional file 3: Figure S1. The br2 allelism test for d2014. [file 12870_2019_2200_MOESM3_ESM.docx]

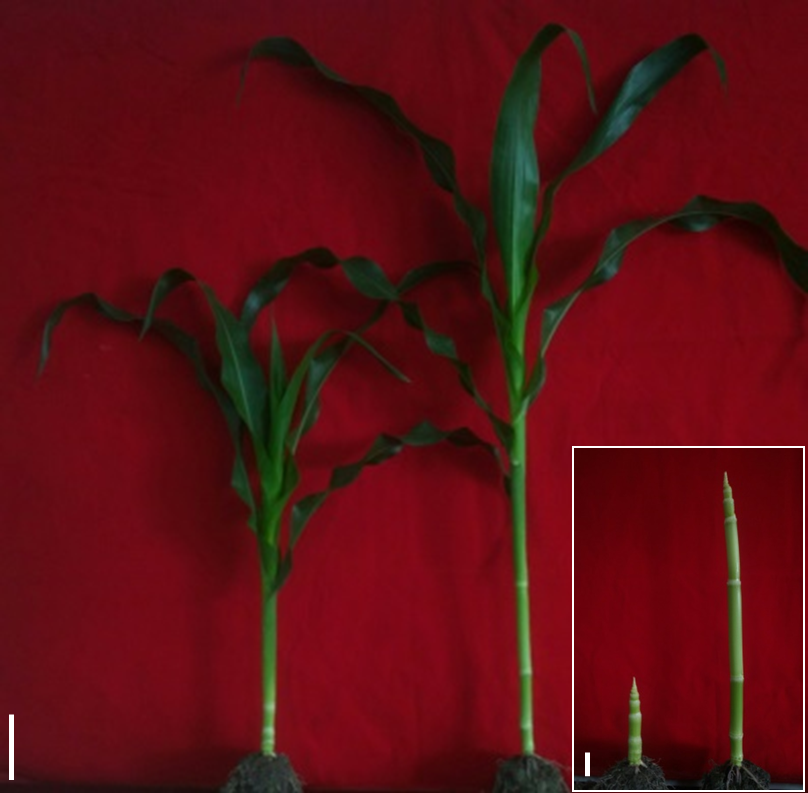


**Fig. S1** The *br2* allelism test for *d2014*. The comparison of plant morphology between hybrid *d2014* × 114F (*br2*) (left) and WT × 114F (*br2*) (right) at the 14-leaf stage, *Bar* = 10 cm; The white box shows the corresponding internode morphology, *Bar* = 5 cm.
