# Supplementary material for: Maize brachytic2 (br2) suppresses the elongation of lower internodes for excessive auxin accumulation in the intercalary meristem region
Source: BMC Plant Biol. 2019 Dec 27;19:589. doi: 10.1186/s12870-019-2200-5 (PMC6935237; doi:10.1186/s12870-019-2200-5)
Supplement: Supplementary file 4 — Additional file 4: Figure S2. Comparison of certain internode length between d2014 and WT at the 12-leaf, 14-leaf, and 20-leaf stages. [file 12870_2019_2200_MOESM4_ESM.docx]

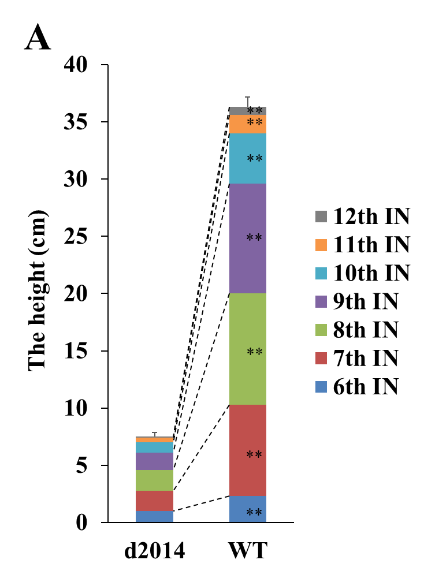

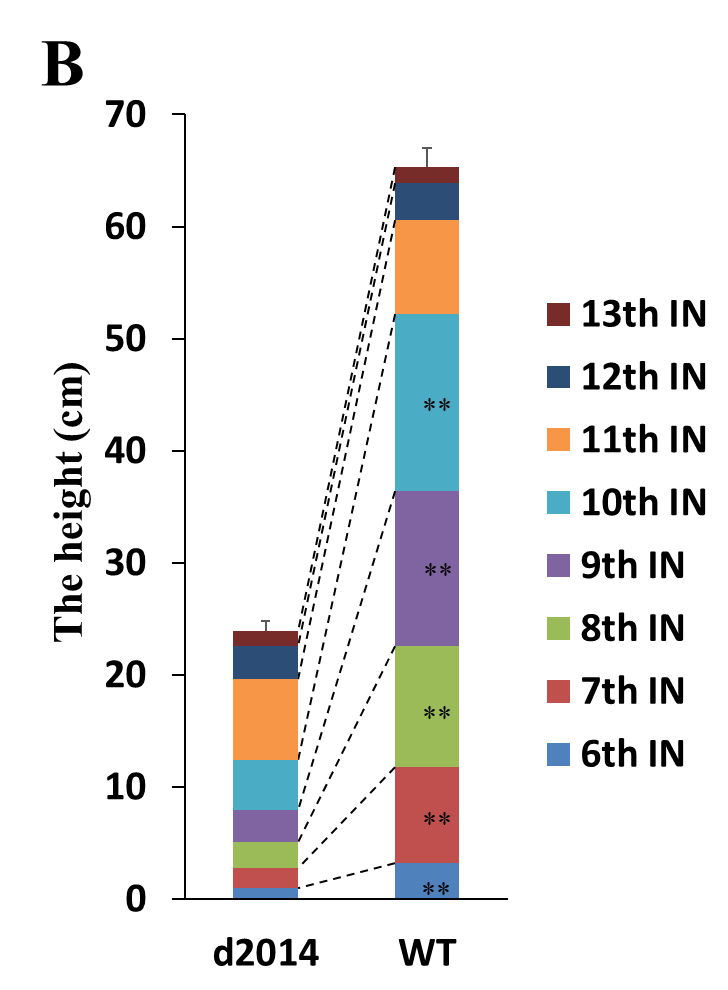

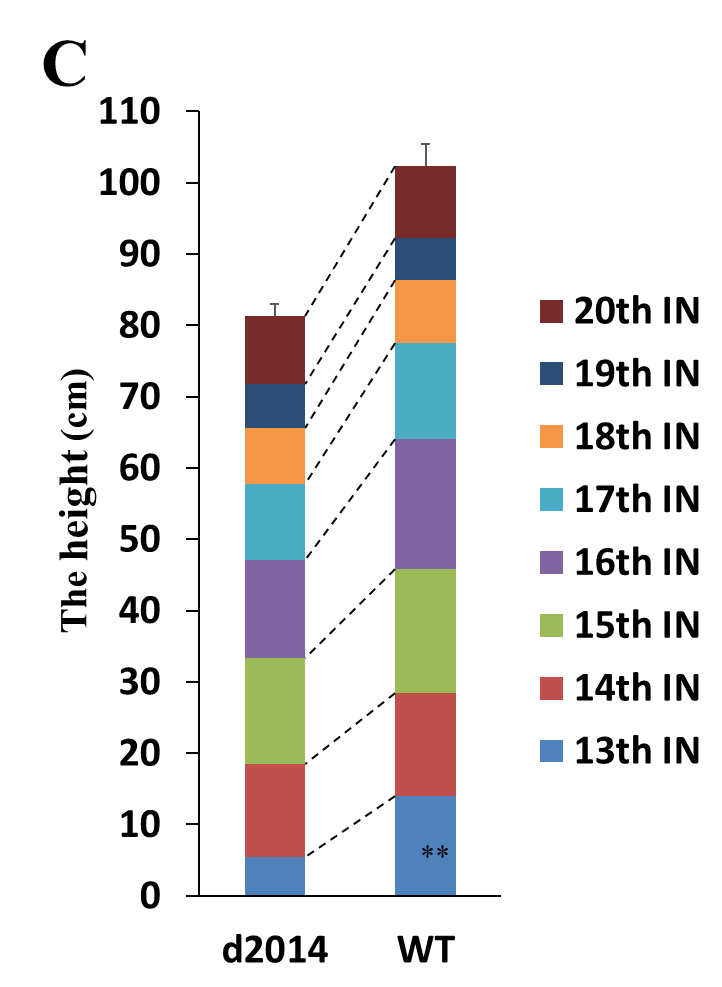


**Fig. S2** Comparison of certain internode length between *d2014* and WT at the different stages. **A** The length of all the visible internodes at the 12-leaf stage. **B** The length of the lower internodes at the 14-leaf stage. **C** The length of the lower internodes at the 14-leaf stage. IN indicates internode; The 13^th^ IN is the ear-internode; ** Significant difference between *d2014* and WT at 0.01 level by *t* test.
