# Supplementary material for: Maize brachytic2 (br2) suppresses the elongation of lower internodes for excessive auxin accumulation in the intercalary meristem region
Source: BMC Plant Biol. 2019 Dec 27;19:589. doi: 10.1186/s12870-019-2200-5 (PMC6935237; doi:10.1186/s12870-019-2200-5)
Supplement: Supplementary file 5 — Additional file 5: Figure S3. Different mutation sites of br2 alleles. [file 12870_2019_2200_MOESM5_ESM.docx]

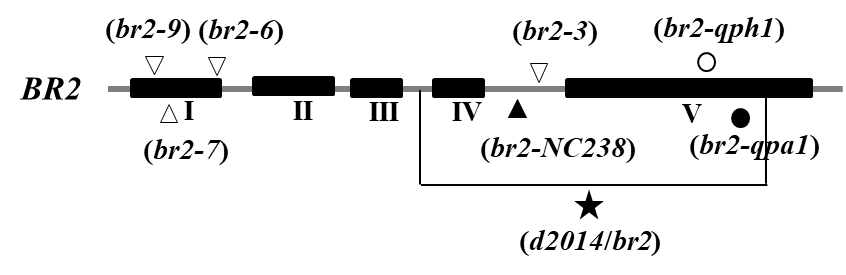


**Fig. S3** Different mutation sites of *br2* alleles. Exons are shown as black boxes; introns are shown as gray lines. *Mu* insertions in *br2-3*, *br2-6*, *br2-7*, and *br2-9* alleles are shown as white triangles (Multani et al. 2003); a novel transposon in *br2-NC238* is shown as black triangles (Balzan et al. 2018); one SNP variant in *br2-qph1* is shown as white roundness (Xing et al., 2015); a 241 bp deletion in *br2-qpa1* is shown as black roundness (Wei et al., 2018); the black pentagram indicates the *d2014*/*br2* allele.
