# Supplementary material for: Maize brachytic2 (br2) suppresses the elongation of lower internodes for excessive auxin accumulation in the intercalary meristem region
Source: BMC Plant Biol. 2019 Dec 27;19:589. doi: 10.1186/s12870-019-2200-5 (PMC6935237; doi:10.1186/s12870-019-2200-5)
Supplement: Supplementary file 6 — Additional file 6: Figure S4. The analysis of protein domains of PGP1-T01 and PGP1-T02. [file 12870_2019_2200_MOESM6_ESM.docx]

**
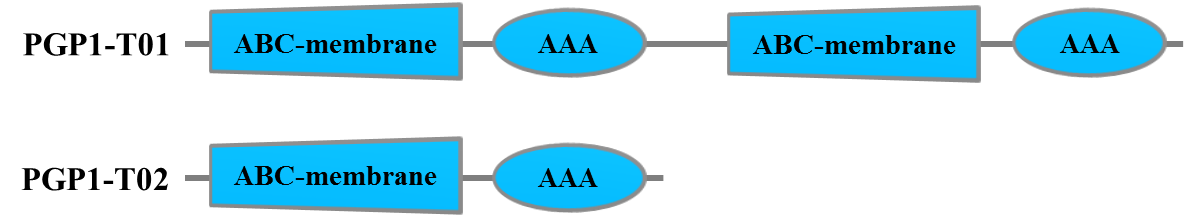
**

**Fig. S4** The analysis of protein domains of PGP1-T01 and PGP1-T02. ABC-membrane indicates TMD domain, while AAA indicates NBD domain.
