# Supplementary material for: Maize brachytic2 (br2) suppresses the elongation of lower internodes for excessive auxin accumulation in the intercalary meristem region
Source: BMC Plant Biol. 2019 Dec 27;19:589. doi: 10.1186/s12870-019-2200-5 (PMC6935237; doi:10.1186/s12870-019-2200-5)
Supplement: Supplementary file 7 — Additional file 7: Figure S5. The morphological characteristics of the stems of d2014 and WT. [file 12870_2019_2200_MOESM7_ESM.docx]

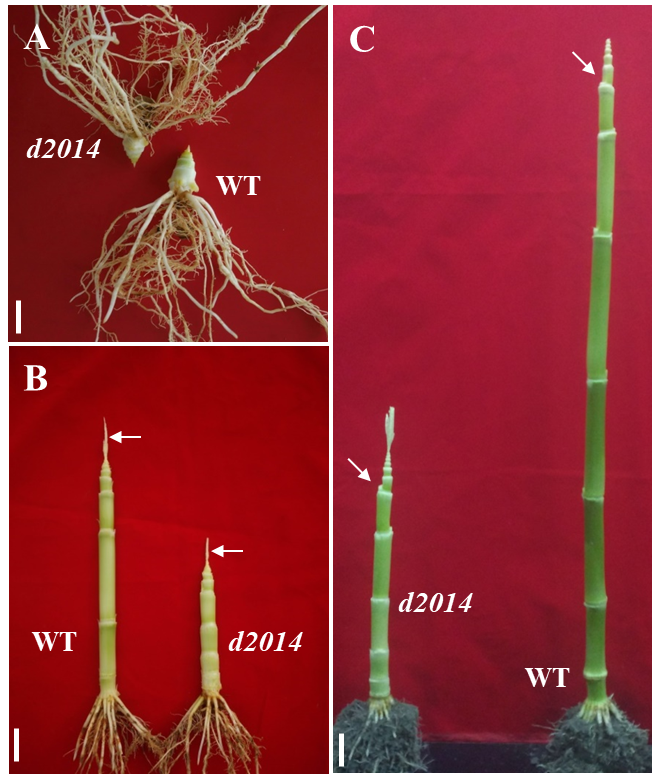


**Fig. S5** The morphological characteristics of the stems of *d2014* and WT. **A** Morphology of the internodes at the 6-leaf stage. The first internode (6^th^ internode) above ground was visible. *Bar* = 1 cm. **B** Morphology of the internodes at the 12-leaf stage. The arrows indicate the visible young tassel. *Bar* = 3 cm. **C** Morphology of the internodes at the 14-leaf stage. The arrows indicate the ear-internode when the upmost AM emerged. *Bar* = 2.5 cm.
